# Respiratory Diseases

**Published:** 1905-03-25

**Authors:** 


### RESPIRATORY DISEASES.

(*Continued from page* 449.)

**Feeding in Tuberculosis.**—Philip[6] advocates the use of raw meat. It has been found that in dogs who were inoculated with tuberculosis an ordinary diet, or one of cooked meat, resulted in progressive emaciation, but a diet of raw meat, or of the juice, actually led to an increase of weight. It is suggested that this arises from the presence of anti-toxic ferments in raw meat which are destroyed even by boiling. In human beings Galbraith found that raw meat caused an increase of nitrogenous retention and of hæmoglobin, while the digestive leucocytosis was sometimes more than doubled. Philip has noted the results in more than 200 consumptive patients, and finds that they quickly develop a liking for it, and improve wonderfully in every respect. He gives finely-minced beef, cold or warmed, to the amount of half-a-pound, two or three times daily, or meat juice, or a soup made with warm milk and minced beef. Burton Fanning,[7] while acknowledging the danger of over-feeding, points out that judicious over feeding is required, for usually the patient is under his proper weight, and the strain of the disease and of the fever requires to be met. Though absolute rest in bed reduces the fever more certainly than anything else, copious food is well borne in moderate fever, and seems to aid in reducing the temperature. He thinks that $4\frac{1}{2}$ ounces of proteid, 5 of fat, and $10\frac{1}{2}$ of carbohydrates are a good diet to commence with for the ordinary consumptive. This is Foster's diet for a healthy man in active life with the addition of 2 or 3 ounces of fat. The meat, too, can be gradually increased to nearly double. The patient is often inclined to take little, but the amount for each meal should be carefully measured, the reason explained to him and persuasion employed to induce him to consume it. Thus the standard diet includes 5 ounces of meat, 2 of butter, 1 egg, 3 pints of milk, $\frac{1}{2}$ lb. of bread, a plate of porridge, besides potatoes and puddings. If weight is not gained and the fever reduced upon this diet a larger quantity is ordered. The addition of bicarbonate of soda to the milk will often aid digestion, and plasmon or casumen can be used to increase the proteid if there is a real difficulty in digesting meat. Arthur Latham agrees in the main with the last writer and emphasises the need of variety in the diet. He finds that larger diets are best borne by those whose weight is sub-normal, and generally that more proteid and fat should be taken than is required by a healthy active individual. When the weekly weighing shows that the normal weight is reached, care should be taken to avoid over fattening. If albuminuria is present, an excess of proteid may easily lead to uræmia, and in high fever and real gastric disorders various substitutes for ordinary food are needful. Though alcohol is not required in many cases, it is often of use in fever and sometimes as an aid to the appetite and digestion, though occasionally it has the opposite effect.

Streptothricosis.—Warthin and Olney [8] show that a lung affection, which may be diagnosed as tubercular phthisis, has in a few cases been actually due to a form of streptothrix. These cases are quite different from actinomycosis, and the organisms are not the same though allied. The disease has been found in various parts of the world, and may be indistinguishable from tuberculosis. The sputa shows a branching mycelial growth staining with carbol fuchsin, and not losing the stain when washed in acids. The clubs or rosettes of actinomycosis are not present, and there are no bacillary or coccus forms. It is important that a thorough investigation should be made of cases when they occur, both as to the behaviour of the organism under cultivation and as to the clinical differences between these cases and those of tuberculous and syphilitic lung affections.

Infection by Flies.—F. T. Lord [9] finds that flies may play an important part in the spread of tuberculosis. They devour sputa and the bacilli multiply in their bodies and are found in their droppings in a virulent state for as long as 18 days. Though they may not be set free into the air, when the fly droppings are undisturbed, still these droppings are often deposited on human food and may thus give rise to intestinal and other forms of tuberculosis. In spite of the rapid increase of the bacilli in the intestines of flies, the insects do not appear to become affected. The writer insists on the importance of protecting from flies all tubercular sputa, or discharges, and also of preventing their access to all food stores.

Bovine Tuberculosis.—Nathan Raw's theory [10] that human beings are subject to two distinct infections, one from their own proper bacilli and the other from the bovine strain is full of interest, and suggests explanations of numerous difficulties. The latter infection, he thinks, comes through milk or meat, and sets up tabes mesenterica, strumous joints and glands in children, miliary tuberculosis, and sometimes a special form of phthisis through the lymphatics and glands, while the bacilli, though apparently the same as in the other group, present great differences in their growth on culture media. About one cow in four of dairy cattle suffers from tuberculosis, but only perhaps one in 20 gives tuberculous milk, which is, however, mixed with the general supply. Breast-fed children never develop abdominal tuberculosis, and it is very rare in India, Egypt, and the East, where cow's milk is very little used, and what is employed is practically free from tuberculosis. On the other hand, ordinary adult phthisis is conveyed by inhalation from one person to another, and rarely affects the joints or the glands, except in late stages. While the bovine bacilli do infect human beings, but in a special and limited way, inoculations of human bacilli grow with difficulty in cattle. Indeed, the German Health Office only succeeded in one case, where the material came from a child suffering from abdominal tuberculosis, which on Raw's theory would be really bovine after all. Some degree of immunity is produced against an attack of one type of tuberculosis by a previous infection with the other. Thus children with strumous glands do not often become phthisical in later life, though phthisis may appear as a late stage in their special disease. Römer finds that a vaccine made from purely human bacilli is the only one which gives cattle complete immunity against bovine tuberculosis, and Raw, on the strength of these observations, is endeavouring to obtain from the serum of tuberculous animals a vaccine to be used in the treatment of phthisis.

Racial Phthisis.—Shrubsall [11] has been investigating the stature, pigmentation, and shape of head in phthisis and other diseases as an indication of racial tendencies among European populations. In this country it would seem that sufferers from tuberculosis are in either sex shorter than the average, though there are also many patients who are tall, but show a deficient chest measurement. There does not appear any special type of skull peculiar to either of the ancient race stocks which is found especially in tuberculosis. Inquiries as to pigmentation disclose a great predominance of phthisis among the darkhaired types, which agrees with its prevalence in just those areas of the country which are inhabited by dark races.

[6] Pract., Jan. [7] Loc. cit. [8] Amer. Jour. Med. Sci., Oct. [9] Boston Med. and Surg. Jour., Dec. 15. [10] Brit. Med. Jour., Oct. 8. [11] Ibid., Dec. 24.

